# UAS Safety Operation – Legal Issues on Reporting UAS Incidents

**DOI:** 10.1007/s10846-021-01448-5

**Published:** 2021-10-27

**Authors:** Anna Konert, Piotr Kasprzyk

**Affiliations:** 1 Institute of Air and Space Law, Dean of the Faculty of Law and Administration, Lazarski University in Warsaw, Warsaw, Poland; 2 Institute of Air and Space Law at Lazarski University, Warsaw, Poland

**Keywords:** Drones, UAS, UAS incidents, UAS safety, UAS regulations

## Abstract

Introduction. This paper examines regulations which govern procedures for reporting incidents other than accidents or serious incidents related to unmanned aircraft system (UAS) operations. The regulations are discussed in the context of available data and the paper included an analysis of them from both a European and national perspective. The goal of the paper is to provide a series of recommendations with regard to the procedures for reporting and analyzing UAS incidents in order to improve the safe integration of unmanned and manned aviation. This article also explores the legal consequences that arise from the midair collision between a UAS and a manned aircraft. Material and methods: The method of study comprises a content analysis of existing legislations. The current doctrine was confronted with existing regulations, documents and materials. Results: The results of the study show that there is a practical problem of objectively identifying operators of a UAS as well as in defining what exactly constitutes an “incident”. It can be reasonably concluded that reporting and analyzing UAS-related incidents allows for the assessment and development of strategies for integrating manned and unmanned aviation. It is worth mentioning that drones and UAS technology requires refinement, especially in technological terms. It is reasonable to take action aimed at raising awareness amongst UAS users of the need to report incidents, as well as engaging UAS users in the investigative process which follows such occurrences.

### 1 Introduction

The rapid progress in the field of modern technologies affects the development of unmanned aerial vehicle systems that are used for military and civilian purposes. There are no technical limitations to the size of unmanned aerial vehicles (UAVs). A drone can either weight just a few hundred grams, or be the size of a Boeing 777. [1]

Drones are used on a large scale. They make it possible to test air quality through the remote measurement of combustion pollutants in the air, save lives in Africa (drones produced by Zipline will support medical transport in Tanzania), and are also used in emergency medical services. The Polish Air Force Institute of Technology has created a drone called AtraxM, which is designed to support rescue operations and air transport. It is characterized by its long mission range (up to 10 km) and a flight duration of up to 40 min. It is perfectly suited to be used as a scout to identify the scene of an incident prior to the arrival of emergency services, check on the number of victims or determine the scale of an accident. It can also carry a first aid kit containing basic dressing materials; an anti-shock kit, blood bags or drugs. Moreover, some countries, including China and Poland, are currently using drones to support their efforts in combating the effects of COVID-19. [1]

The UAV and UAS markets have been showing steady, increasing growth year after year in Europe. Some of the world's top drone service providers are based here, and their results are proof of continuous and considerable market growth. Some forecasts predict that the European drone market will more than double in size between the years 2018 and 2024 [2]. Others predict that revenues generated from commercial drone use in Europe will grow from USD 251 million in 2020 to USD 3 billion in 2025. Different estimates see the region's drone market bringing in EUR 10 billion annually by 2035, and over EUR 15 billion annually by 2050. [3].

✉ Anna Konert
a.konert@lazarski.edu.pl

Piotr Kasprzyk
piotr.kasprzyk@lazarski.pl

1 Institute of Air and Space Law, Dean of the Faculty of Law and Administration, Lazarski University in Warsaw, Warsaw, Poland

2 Institute of Air and Space Law at Lazarski University, Warsaw, Poland

Different labels are applied to unmanned aircraft systems. [4, 5]. The most commonly used terms for drones are model aircraft, pilotless aircraft, autonomous aircraft, unmanned aircraft (UA), unmanned aircraft system (UAS), unmanned aerial vehicle (UAV), remotely piloted aircraft system (RPAS), remotely piloted aircraft (RPA), or remotely piloted vehicle (RPV). Another common term, alongside "drone", was the term *pilotless aircraft*. It had been used in the Paris Convention of 1919, but was later changed ("aircraft… capable of being flown without a pilot") in the Protocol of 1929. Subsequently, this provision was repeated in Art. 8 of the Convention on International Civil Aviation signed in Chicago on December 7, 1944. - the Chicago Convention (Journal of Laws of 1959, No. 35, item 212, as amended) (*"aircraft capable of being flown without a pilot"*). The term "pilotless aircraft" can be understood in a variety of ways depending on how we define the term "pilot". The first approach would be to apply the term only to onboard personnel. The second approach would be to use the term to also describe crew involved in the flight but not present onboard (*remote pilot*). Finally, it could also signify personnel that sets an aircraft in motion but has no further control over it (i.e. *"launchers"*) [6]. According ICAO, a pilot-in-command is "the pilot responsible for the operation and safety of an airplane in flight" (Annex 6 to the Chicago Convention). In Poland, an aircraft's crew consist of people designated by the aircraft operator to perform tasks onboard the aircraft while it is in flight. A manned aircraft in flight must have an appointed aircraft commander, hereinafter referred to as "commander", so it is clearly emphasized that a pilot is present on board. The obligation to appoint a commander means that "a flight cannot take place without a designated pilot-commander" [7]. ICAO's definition seems to also cover piloting an aircraft remotely (*remote pilot*). While some claim that the literal wording of Art. 8 of the Chicago Convention does not include RPA, its functional interpretation – which takes the subject and purpose of the regulation into account – does. [8] Nonetheless, there is no doubt that operations without a pilot (*pilotless aircraft*) are autonomous (*autonomous aircraft*) i.e. they do not allow for pilot intervention in flight management.

Nowadays, the term "*unmanned aircraft*" (UA) is gaining popularity and is widely used. It has been officially recommended by ICAO and adopted by the EU and EASA. ICAO uses the term UAV, but as a broad definition including autonomous drones. However, it focuses on regulating only those that fall into the RPA category, since the presence of a pilot is a guarantee of safety. [1, 9]

In this paper, the Authors will use either unmanned aircraft systems (UAS) or drones.

It should be underlined that an unmanned aerial vehicle meets the technical requirements necessary for it to be considered an aircraft, as it is a device capable of maintaining flight through the use of forces other than those that bounce air off the ground (Annexes to the Chicago Convention). Therefore, in the scope of public law, a drone is an aircraft intended for use without a pilot on board. In the absence of specific regulations concerning unmanned aircraft, it should meet the requirements for regular aircraft, which in practice is not possible. The century-old system of regulations in the field of civil aviation safety (at an international level, and consequently also at a European and national level) is based on the assumption that there is a flight crew on board, directing or supervising a flight. [1, 10]

Subject literature indicates that only 30% of currently existing regulations in the field of aviation safety are fully applicable to unmanned aircraft, that 54% are partially applicable once necessary changes have been made, and that 16% are not applicable at all. [11]

A growing number of drone operations have increased the number of dangerous events involving UAS, and this is not just incidents and airprox with UAS reported by aircraft crews in recent years. This also includes accidents that result in damage or destruction of UAS. Meanwhile, rules for investigating air accidents, which have been developed over the past few decades, remain virtually unchanged [12].

Following ICAO recommendations, some countries require that safety investigation authorities investigate accidents involving some types of drones.

In the USA, for example, the NTSB is required to investigate an accident involving UAS [13] if one of the following conditions are met:

1) *Any person suffers death or serious injury; or*
2) *The aircraft has a maximum gross takeoff weight of 300 pounds or greater and sustains substantial damage.*

Since 2010, when this rule was adopted, there have been only five reported UAS accidents subject to the NTSB investigation. [14]

The UK's AAIB is another example of adopting a general policy which states that accidents and other serious UAS incidents must be investigated if a UAS has been allowed to operate with permission from an aviation authority, which is required for:

1) UAS weighing over 20 kg (obtaining a permit to fly requires that the UAS is subjected to a technical assessment),
2) certain activities, such as BVLOS flights (operational authorization).

The UK's AAIB has received completed accident report forms on 36 accidents involving UAS since February 2015. [15].

Adopting the policy on investigation of the UAS accidents is challenging, especially in regard to accidents, where the safety of manned aviation was not endangered. Such policy shall address the following problem: *to what extent is it desirable or practical to investigate accidents and incidents of UAS of different sizes?* [16] Resources to investigate aviation accidents are limited, and safety investigation authorities are focused on the safety of the commercial transportation system. It must be also noted, that although accident investigation process is extremely mature in manned aviation, some aspects of the accident investigation process must be improved or developed with regard to UAS accident investigation [12].

Another aspect is that the investigation of aviation accidents and major incidents is a reactive approach to aviation safety. Accidents and serious incidents are investigated to avoid similar occurrences in the future. This occurs through the investigation of such events, i.e. determining the causes (both root causes and contributing factors), and if necessary, issuing safety recommendations.

Along with the development of aviation and safety management, it was necessary to supplement this traditional approach to safety with proactive solutions. This approach is associated with the development of so-called safety management systems (SMS) in aviation organizations. One of the pillars of SMS is the reporting (mandatory and / or voluntary) of all occurrences that may affect aviation safety, not just accidents or serious incidents. Thanks to this, it is possible to identify potential threats at the earliest possible stage, which allows for corrective action to be taken to eliminate these threats before an accident or a serious incident occurs. [17]

The rules for reporting civil aviation occurrences at EU level, have been harmonized in the provisions of EU Regulation No. 376/2014 on the reporting, analysis, and follow-up of occurrences in civil aviation. [17]. EU Regulation No. 376/2014 applies to "*any organizations providing aviation products and/or which employs, contracts or uses the services of aviation professionals*".

### 2 UAS Safety Operations Regulations

The ease of access to unmanned aircraft, their technical capabilities and a wide scale of application, make them increasingly popular in recent years, which is a big challenge when it comes to the safety level of air operations not only of unmanned aerial vehicles, but also manned aircraft operations that use the same airspace. In order to maintain an acceptable level of safety, the drone operator should familiarize himself with the regulations containing the conditions for the safe performance of operations. It is extremely important because the conscious use of unmanned aerial vehicles is the basis for caring for one's own safety and that of others.

Aviation safety regulations are complex, multiplanar and shaped on many levels. They are based on various sources of law (international, EU and national) and have different subjective and objective scopes of application. In addition, they are highly detailed in their reference to organizational, technical and operational issues.

Regulations on the safety of drones operations must take into account the characteristics and properties of drones. To do so, it is necessary to take into account the basic differences between manned and unmanned aircraft, through a certain level of generalization. The differences should be perceived from the perspective of the primary goal of aviation safety regulations, which is as follows: to achieve at least the same level of safety for unmanned aviation operations as the one achieved in manned aviation. If we look at aviation safety as a situation in which the risks associated with certain activities are reduced to an acceptable level and controlled, then these risks must first be identified.

On December 31, 2020, the European Union will standardize drone regulations across the continent and the new rules replace each EU state's existing laws.

This new EU regulatory framework (Commission Delegated Regulation (EU) 2019/945 and Commission Implementing Regulation (EU) 2019/947) [18, 19] includes both technical and operational requirements for UA, their operators and pilots. They define UA capabilities, types of operations, and label these into three broad risk-based categories: open, specific, and certified. These three categories of operations are based on their levels of risk involved and each adopts a varied regulatory approach. [3] The open category covers low risk operations. Safety is ensured by compliance with the relevant requirement. No authorization is required. The specific category is related to more risky operations. Safety risk assessment is a prerequisite to obtain an operational authorization from the competent authority. In the certified category safety should be ensured by UA certification and remote pilots licensing.

Another important issue to maintain an acceptable level of safety, is reporting UAS occurrences.

Such occurrences should be reported by aviation professionals. And reporting systems are usually part of the aviation organization safety management system.

It must be noted that Commission Implementing Regulation EU 2019/947 allows some drone operators i.e. legal entities, to be certified operators. So called Light Unmanned Aircraft Certificate LUC will entailed the operator to authorize its own operations in the specific category. And one of the main requirements for such certification is that the operator shall sh*all establish, implement and maintain a safety management system corresponding to the size of the organisation, to the nature and complexity of its activities, taking into account the hazards and associated risks inherent in these activities (UAS.LUC. 030).* Safety reporting and internal investigation are obvious part of such system.

However, the abovementioned EU implementing rules on drones are not setting up any new requirements for reporting or analyzing drone related safety occurrences.

Therefore, general aviation rules for reporting civil aviation occurrences applies. At EU level those rules were created by the EU Regulation No. 376/2014 on the reporting, analysis, and follow-up of occurrences in civil aviation.

### 3 EU Regulation 376/2014

The most important assumptions of the EU Regulation No. 376/2014 are as follows:

1) aviation professionals (pilot in command, maintenance personnel, ATC, etc.) are obliged to report occurrences which may represent a significant risk to aviation safety;
2) an occurrence is a very broad term. It covers both accidents and serious incidents (subject to investigation by independent commissions), as well as much less serious (but much more frequent) incidents and so-called other occurrences that affect or may affect the safety of aerial operations. These types of events have been defined in detail in the implementing regulation, adopted by the European Commission [13];
3) organizations employing, contracting or using the services of aviation professionals are required to put in place systems to facilitate the collection of both mandatorily and voluntarily reportable occurrences. For organisations subject to safety management systems requirements, it is understood that such systems should be part of the organisation SMS;
4) after the occurrence has been reported within an organization, it is also forwarded to the competent authority for the purpose of entering data from it into a European database, ECCAIRS (*European Coordination Center for Accident and Incident Reporting Systems* - https://eccairsportal.jrc.ec.europa.eu),
5) if the organization has decided to take corrective action as a result of occurrence analysis, the competent authority should be notified of the implemented action,
6) reporters are subject to legal protection, which means that the organization that employs them, as well as the competent authority or aviation authority, may not take negative action against them (or persons participating in the event) unless the incident was caused intentionally or through gross negligence,
7) based on data on safety-related occurrences, aviation organizations take corrective actions following a particular incident or group of incidents; analyses and actions may be also taken at the Member State or EU level; [20]

EU Regulation 376/2014 applies to safety occurrences involving aircraft for which EU competencies arise from the so-called Basic Regulation. This rule, written in art. 3 s. 2 of EU Regulation No. 376/2014, was modified when the new Basic Regulation, i.e. EU Regulation No. 2018/1139, was adopted. [18] Extending the EU's competences to unmanned aerial systems would provoke a necessity to report all safety-related occurrences involving all categories of UAS. This is why the applicable subjective scope of Regulation No. 376/2014 has been limited when it comes to UAS-related occurrences. Art. 3 s. 2 states as follows:

*However, this Regulation shall not apply to occurrences and other safety-related information involving unmanned aircraft for which a certificate or declaration is not required pursuant to Article 56(1) and (5) of Regulation (EU) 2018/1139, unless the occurrence or other safety-related information involving such unmanned aircraft resulted in a fatal or serious injury to a person or it involved aircraft other than unmanned aircraft.* [20]

Primarily, the focus here is complex UAS that are either produced within an existing certification system or are part of a manufacturer declarations system. They are a category provided for in the new Basic Regulation but not regulated in the delegated act published by the European Commission in 2019. EC Regulation No. 2019/945 [19] orders production of open category UAS to comply with CE (Conformité Européenne) compliance regulations and refers production of special category UAS to technical requirements specified in the process of issuing operational authorizations. In other words, the Basic Regulation set up the legal framework that will allow the EC to regulate the complex UAS production in the future (as a certification or declaration system). But, as of now, no implementing rules on the production of the complex UAS have been published. [20]

Therefore, the obligation to report an occurrence involving UAS is interrelated with two situations:

1) an incident involving a UAS which resulted in death or serious injury,
2) a manned aircraft took part in the occurrence involving a UAS.

It must be stressed that the obligation to report occurrences under EU Regulation 376/2014 is independent and complementary to the obligation to report accidents and serious incidents to the safety investigation authority. It means, that in the event of an accident or a serious incident, notification is also subject to the EU Regulation No. 996/2010 on the investigation and prevention of accidents and incidents in civil aviation. [20, 21]

## 4 UAS Incident Statistics

### 4.1 EU Statistics (ECCAIRS)

The provisions of Regulation No. 376/2014 had been in force since November 2015. When discussing available statistics related to the safety of UAS operations, it is justified to focus on the time period between 2015 and 2018.

One of the first sets of statistics concerning UAS occurrences on the EU level was published in the EASA Annual Safety Report (ASR) for 2016, based solely on ECCAIR data. According to this data, approx. 400 drone occurrences were noted in 2015 Figure 1-16.

Based on ECCAIRS data found in the Annual Safety Report for 2017 (published by the Polish CAA), the total number of notifications in ECCAIRS database regarding UAS (excluding "undefined") looked as follows: approx. 400 (2015), approx. 800 (2016), approx. 1150 (2017).

A rapid increase in reported incidents in the following years is clear (increases of several hundred percent). However, it should be noted that the vast majority of these incidents (reported in ECCAIRS) have been classified as incidents or occurrences.

It should be borne in mind that data from the ECCAIRS database may be subject to some systemic imperfections since it is reported at a Member State level. Sometimes, an occurrence gets re-classified after it's been reported. These tend to happen during the report follow-up procedure when more information is available.

It is, however, possible to provide the total number of UAS-related incidents that were reported to the ECCAIRS database between 2015 and 2018. This data was obtained at the end of 2019 from the PL CAA as a response to a motion submitted by the authors of this article on the basis of Regulation No. 376/2014 (Letter of the PL CAA no LBB-2/811–0006/19.).

At an EU level, in the ECCAIRS database, between 2015 and 2018, there were, among others:

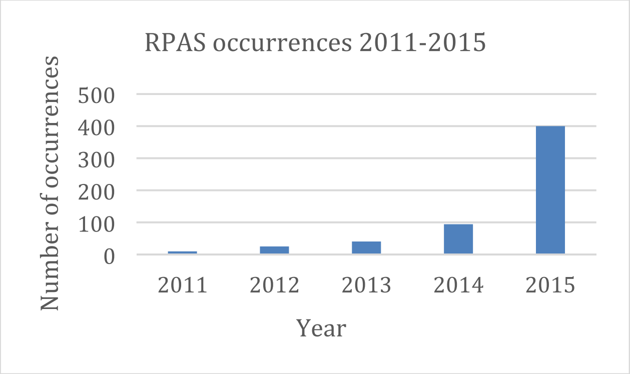

**Fig. 1** EASA ASR for 2016

- 104 accidents involving UAS (with only one accident concerned a collision of a UAS with a manned aircraft),
- 91 serious incidents involving UAS,
- 3301 incidents involving UAS,
- 424 other occurrences involving UAS,
- 594 other reports (so-called non-determined) involving UAS.

However, the vast majority of reported serious incidents and incidents were related to incidents involving both unmanned aircraft and manned aircraft. This is due to, as it was mentioned earlier, the specifics of the incident reporting system, which imposes the obligation to report such incidents on manned aviation personnel.

Anyhow, it can be assumed that the number of official reports in this database does not correspond to the number of all reported incidents involving UAS at national levels. These discrepancies are illustrated by other EASA statistics.

For example, under the so-called Drone Collision Task Force Report (2016), incident statistics from the ECCAIRS database have been supplemented with additional data from EASA and some national aviation authorities. In this context, the total number of incident reports for 2015 amounts to approx.. 500 (25% more than in the ECCAIRS).

On the other hand, in the following EASA annual safety report for the year 2017, data on incidents involving drones were based not only on ECCAIRS but also on other reports from EASA or certain national supervising authorities. In this respect, the total number of incidents for 2015 is approx.. 600 (200 more than in the EASA ASR for 2016).

Unfortunately, for reasons unknown, EASA did not publish any further data on UAS incidents in subsequent years. However, if – to a large extent – we assume that the officially reported number of incidents in the ECCAIRS database involving drones should be increased by about 30%, it should be assumed that the total number of incident reports on an EU / EASA scale was approx. 1200 in 2016 and approx.1600 in 2017.

Some more detailed data can be also found in the EVAIR voluntary reporting system, run by the EUROCONTROL (https://www.eurocontrol.int/service/eurocontrol-voluntary-atm-incident-reporting). This system is of voluntary nature, and its scope is broader then ECCAIRS, as EUROCONTROL is not an EU agency.

Again, we can observe a sharp increase in reported UAS incidents on a European scale, rising ever since 2015. Reports on UAS incidents from EU member states and the USA confirm a similar trend. [20]

### 4.2 National Statistics

Publicly available data on events / reports involving UAS can be found in documents published by the UK CAA.

Fig. 16 the Drone after collision.

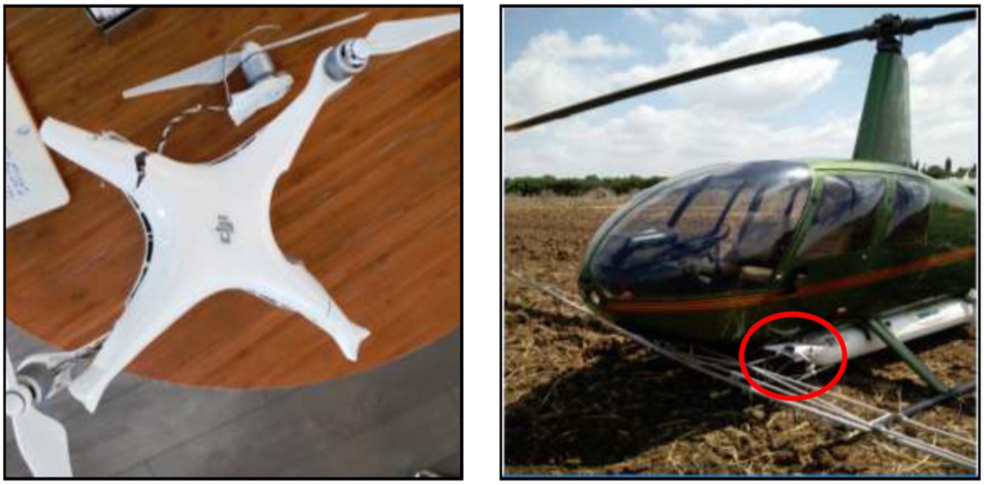

**The Drone after collision** **The drone impact location**

The UK CAA 2016 Safety Report states that about 300 UAS reports were noted in 2016, 87 of which were reported as so-called airpox – which is a UAS in dangerous proximity to a manned aircraft (UK Aviation Safety Review for 2016, CAP 1595).

It is worth noting that the vast majority of UAS flight observation reports concerned operations at altitudes below 4000 ft, with the vast majority being operations below 2000 ft (approx. 700 m).

According to press information published by the German ANSP (DFS) [22], pilots reported the following number of UAS incidents in Germany:

However, the most detailed statistics on drone-related incidents reported by pilots / controllers are published by the FAA (USA). Nearly 200 events per month involving UAS are currently reported via the Aviation Safety Reporting System (NASA ASRS).

Data available for 2017 and 2018 contains statistics (data published with report descriptions) indicating over 200 reports per month:

Also, available data for previous years confirm the upward trend in drone incident reports (2015). [23]

It should be clarified, however, that in addition to the drone incident reporting system for flight crews, there is also a drone incident reporting system in the US that applies to drone operators. Under provisions of PART 107 (§107.9), UAS pilots are obliged to report to the FAA all events that result in

(a) *Serious injury to any person or any loss of consciousness; or*
(b) *Damage to any property, other than the small unmanned aircraft, exceed $500*

No similar obligation has been introduced at an EU level. [20]

### 5 UAS Statistics Anlyses (Examples)

Some of the statistical data are analysed by the safety authorities or regulators. The results of such analyses can be found in published documents on drone safety.

The first example may be the UK CAA document on the safety of UAS operations (Drone Safety Risk: An Assessment, CAP 1627). It provides more detailed statistics on reported UAS flight events for the years 2016 and 2017, which includes information about the reported height at which a pilot / controller observed a UAS.

The majority of sightings took place below 2.000 ft.

Another example is a USA DOT report of 2016 on the FAA oversight for UAS. [24].

At the EU level, EASA published a similar analysis.

Distribution of RPAS occurrences – actual aircraft altitude vs distance to drone can be found in the Drone Collision Task Force Report, published 2016.

| EU | Accidents | Serious Incidents | Incidents | Occurrences | Undefined | TOTAL |
|---|---|---|---|---|---|---|
| 2015 | 12 | 9 | 336 | 52 | 14 | **423** |
| 2016 | 17 | 20 | 702 | 65 | 98 | **902** |
| 2017 | 17 | 17 | 999 | 121 | 126 | **1280** |

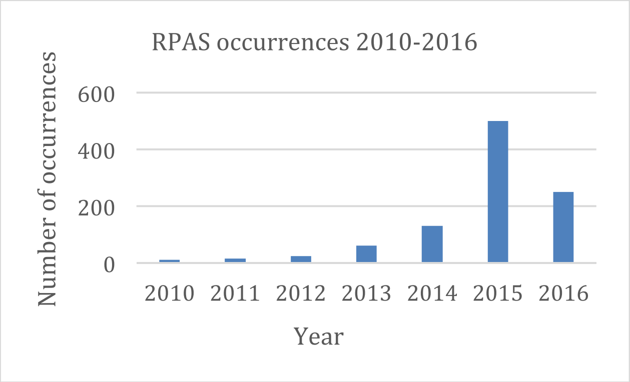

**Fig. 3** EASA Drone Collision TF Report (2016)

Distribution of RPAS occurrences – aircraft altitude vs distance from drone at the time of detection for 2010–2016 was published in the EASA ASR for 2017.

Those analyses, provided by the safety regulators or public authorities relies on the statistical data from the specific reporting systems. It means that those data are subject to a number of uncertainties (i.e. it involves assessment of UAS altitude rather than precise measure).

What is more important, those analyses are based on different data collected by different reporting systems. The key issue is, therefore, the lack of consistency across the states (regions) and their respective database [see ex: 17] As a result, any comparison between national (or regional) reporting systems, makes sense only for high-level analysis.

Available statistics on drone-related occurrences allow observing some general trend. The vast majority of notified occurrences (under national or regional notification systems) are those events, where pilots are reporting a near miss with a UAS or object similar to a UAS. Analyzing different types of UAS-related events, for example, the reported near misses between drones and manned aircraft, allows for identifying systemic problems related to the violation of airspace. What can be easily explained, as rules of the air or airspace design were developed for manned aviation. Therefore, the absence

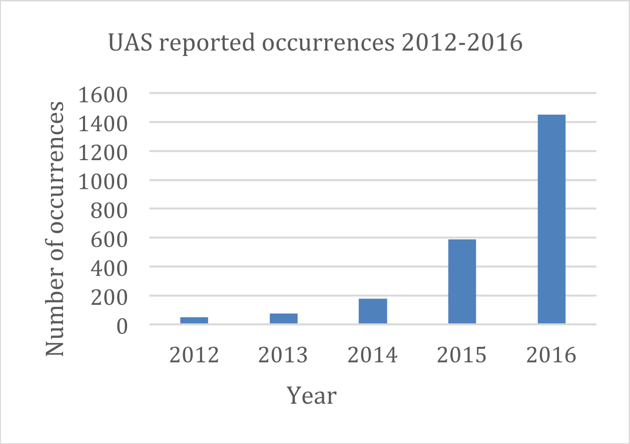

**Fig. 4** EASA ASR of 2017

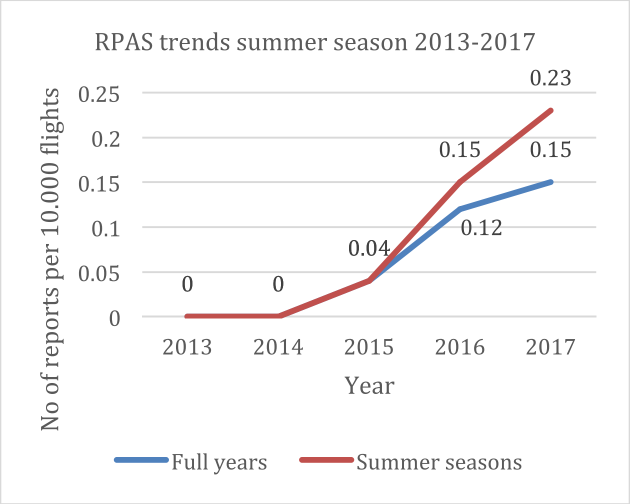

**Fig. 5** EVAIR Bulletin No 20

of a clear and harmonizes legal framework for drones with regard to the airspace does not allow to integrate of UAS operations. [20]

### 6 Collision with Manned Aircraft (Examples and Discussion)

What happens when a drone hits the wing of an airplane?

In 2017 a research team from the Alliance for System Safety of UAS through Research Excellence (ASSURE) released a report on the effects of a collision between a drone and an airplane. The test was made with two different types of drones on two types of aircraft through computer modeling and physical validation testing. The results of the study shows that drones that collide with large manned aircraft can cause more structural damage than birds of the same weight for a given impact speed. Moreover, it was suggested that manufacturers should adopt "detect and avoid" or "geo-fencing" capabilities to reduce the probability of collisions with other aircraft. (The complete report is available at http://www.assureuas.org/projects/deliverables/sUASAirborneCollisionReport.php).

In 2018 Scientists from the Research Institute of the University of Dayton (Ohio, USA) together with specialists from the UAS Training and Certification Center at Sinclair College National made a crash tests of a DJI Phantom 2 drone

| Year | Incidents |
|---|---|
| 2017 | 88 |
| 2018 | 158 |

**Fig. 6** number of UAS incidents in Germany.

| Year | Q1 | Q2 | Q3 | Q4 | Total |
|---|---|---|---|---|---|
| 2018 | 452 | 785 | 671 | 404 | **2312** |
| 2017 | 425 | 635 | 660 | 404 | **2124** |

**Fig. 7** number of reports.

with a Mooney M20 wing. The tests consisted of placing the DJI Phantom 2 drone in a long pipe that can launch (using compressed air for this purpose) various objects at a precisely defined speed. The speed of the DJI Phantom 2 drone collision with the wing during the tests was 238 Mph, which is about 383 km / h. This is exactly the same as the cruising speed of this aircraft (237 Mph), so such a collision could occur if the Mooney M20 plane crashed into the DJI Phantom 2 hanging in the air. The DJI Phantom 2 drone sticks completely into the wing, penetrating it inside and destroying its structure. [25]

According to a FAA report, pilots reported coming in close proximity to approx. 800 drones between April and June 2018 alone. Those sightings have resulted in a few close calls and at least one significant collision.

Drone-related occurrences also involve collisions with manned aircrafts. Until recently, available studies never discussed such events and even when they did, they only mentioned collisions between manned aircraft and the so-called flying model aircrafts, such as remotely controlled manned aircraft replicas used for recreational purposes.

Then, in 2016, EASA's study entitled Drone Collision Task Force Report pointed to an accident from 1997 involving a motor glider with two people on board and a 10 kg model aircraft with a wingspan of 2.4 m. The collision resulted in the glider hitting the ground and killing both the pilot and the passenger.

A UK AAIB annual report from 2018 included two airborne collisions that took place in 2015. Interestingly enough, both accidents involved so-called ultralight aircraft (manned) and models (unmanned) of a very small mass (from half a kilogram to two kilograms). This is significant information as in most countries (including Poland), the rules regarding ultralight aircraft flights are quite liberal. For example, they allow for carrying out flights in areas other than airports or registered airstrips. The accidents mentioned above resulted in

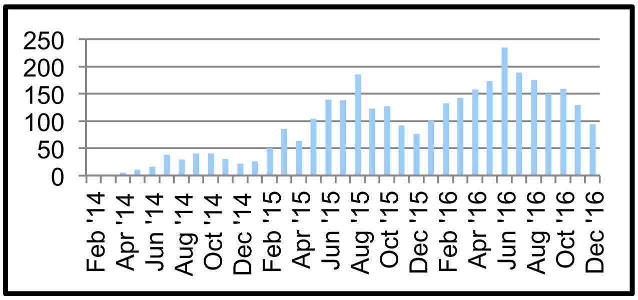

**Fig. 8** J. Guzetti [14]

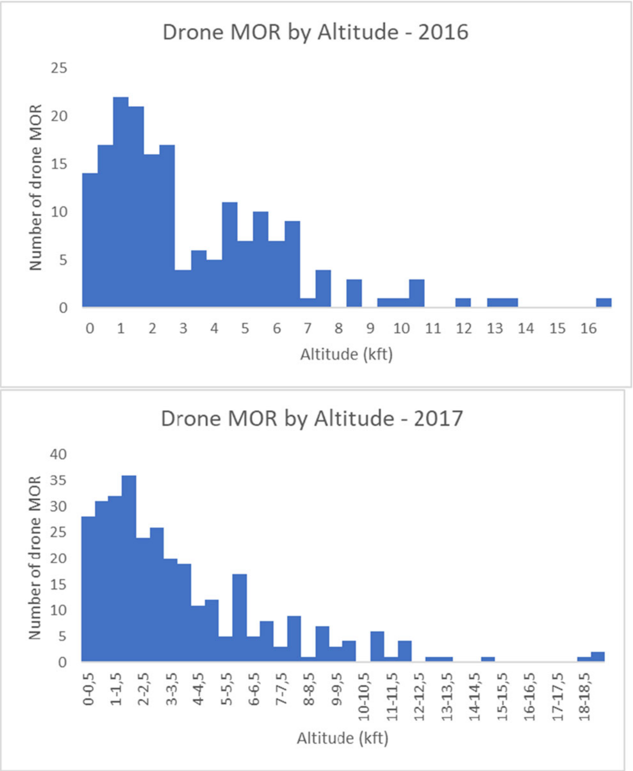

**Fig. 9** Drone Safety Risk: An Assessment, CAP 1627

the models being completely destroyed and the aircraft requiring minor or major repairs.

In recent years, we have come to see an increasing number of incident reports on collisions between manned aircraft and UA other than models. Some of these reports are mentioned below:

1) Drone collides with Beechcraft King Air BE100 (Canada, 2017)

On October 12th, 2017, a plane with a 2-man crew and 6 passengers on board collided with a UA as it descended to land at Jean Lesage International Airport in Quebec, Canada. The collision took place within the airport's controlled

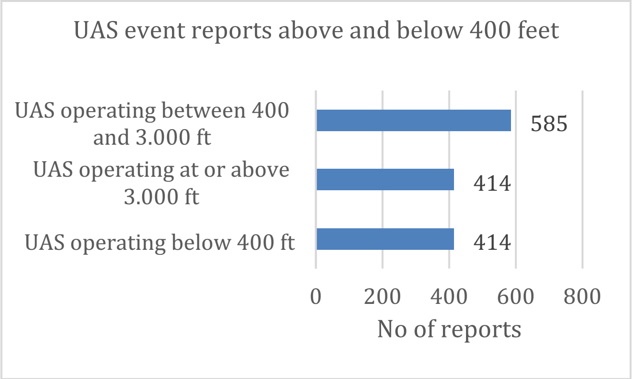

**Fig. 10** J. Guzetti [14]

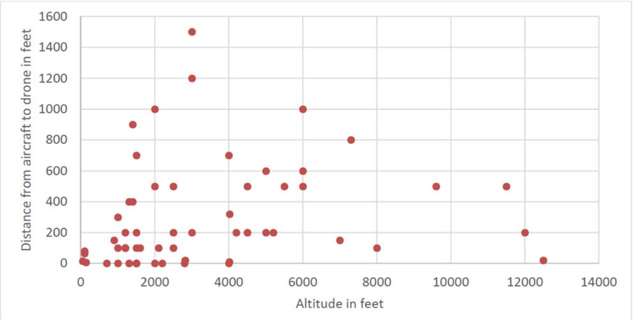

Fig. 11 EASA Drone Collision TF Report 2016

airspace, where the UA was not allowed to fly. As a result, the drone was destroyed and the plane sustained minor damage to one wing.

2) DJI collides with UH60 helicopter (USA, 2017)

The collision between a DJI drone and a UH60 military helicopter occurred on September 21st, 2017 over the New York Bay. As a result, the drone was destroyed and the helicopter's main rotor blade was slightly damaged.

According to the available NTSB report on the matter, the drone operator performed the flight beyond his visual line of sight and was unaware of the applicable air traffic rules, which caused the accident.

3) Drone collides with GC2 helicopter (Switzerland, 2018)

On May 25th, 2018, a helicopter flying towards Locarno airport collided with a drone, resulting in damage to the helicopter's rotor blades. Fortunately, the helicopter reached the airport and landed safely. It was found that the drone pilot was operating within a restricted space as the area around the airport was considered a no-fly zone (5 km). The drone was destroyed.

4) Phantom4 collides with R44 helicopter (Israel, 2018)

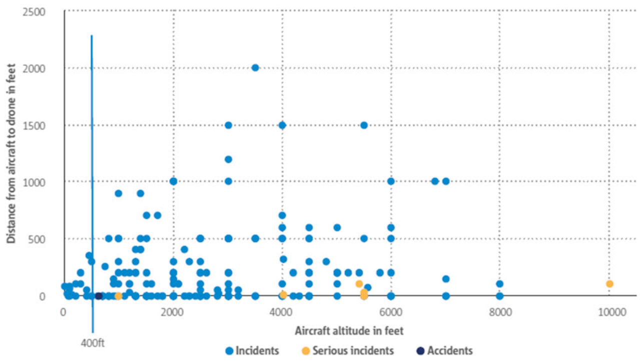

Fig. 12 EASA Aviation Safety Report 2017

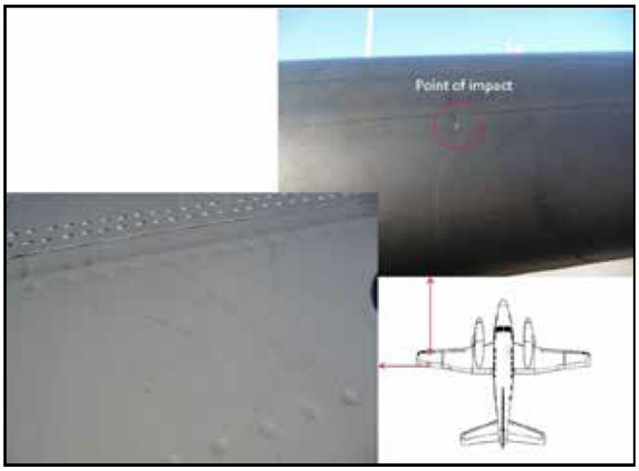

Fig. 13 damage to the aircraft

On August 14th, 2018, a Phantom 4 drone collided with a civilian R44 helicopter performing a crop dusting flight. The drone was destroyed and the spraying system installed on the helicopter was damaged.

An Israeli commission report showed that the drone operator was properly licensed to perform a photo mapping operation of a construction site located near the farmland. However, he didn't notice the helicopter in time and the two collided despite the drone having its emergency landing system activated.

Manned aircraft involved in these accidents sustained only minor damage, which does not allow for the occurrences to be qualified as air accidents subject to investigation (only serious damage can be the reason for a mandatory investigation). However, the UA involved were completely wrecked. From a formal point of view, they should have been treated like any other destroyed aircraft, although they were just small and light drones. Nevertheless, the occurrences were classified as incidents or serious incidents and not as airborne accidents.

It is easy to notice that there is a problem with applying the traditional definitions of an aviation accident, serious incident and incident to occurrences involving UA. In the abovementioned cases, the planes and helicopters involved suffered minor damage so the situations were not classified as accidents. But on the other hand, if we began recognizing drones as aircraft, then they should have been.

Here, it should also be mentioned that occurrences in which only a UA is destroyed and which result in an investigation are considered accidents.

For example, NTSB data shows that between 2010 and 2017, the following accidents involving damaged UA were investigated:

1. May 1, 2015 - experimental UAS Titan Solara crashed after take-off. Probable cause was structural failure of

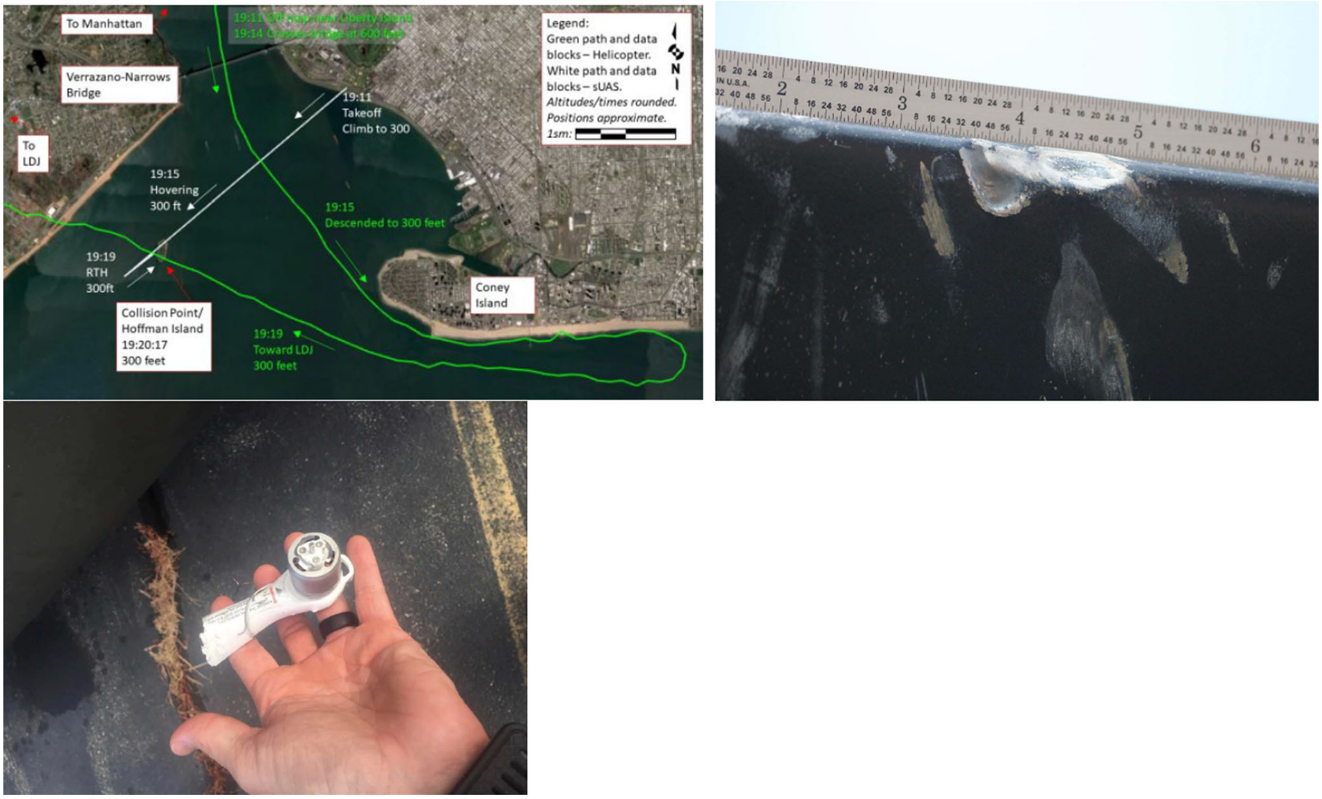

Fig. 14 Pictures of the flight path, the damaged blade and a piece of the drone. Source: NTSB

the left wing due to an overspeed condition caused by a sudden thermal blast;
2. January 27,2014 - MQ-9 Predator operated by the US Customs and Border Protection was substantially damaged following intentional controlled ditching of the aircraft due to failure of the starter/generator;
3. July 26,2013 - SIERRA UAS operated by NASA in research mission in Alasca impacted ocean ice probably due to loss of power caused by icing;
4. May 10, 2013 - MQ-9 Predator, operated by the US Customs and Border Protection, experienced a bounced landing and runway excursion;
5. December 20, 2011 - experimental Meridian UAS, operated by a University, crashed on final approach probably due to aerodynamic stall induced by an inadvertent autopilot Home command.

Large number of final reports on the UAS accident investigation was made available by the UK AAIB, and the respective summary is provided in the AAIB Annual Safety Review 2018 (AAIB Annual Safety Review 2018). It shows that 36 accidents involving UAS were reported in the period 2015–2018. Most of the accidents were related to technical issues, mainly the loss of control of the UAS.

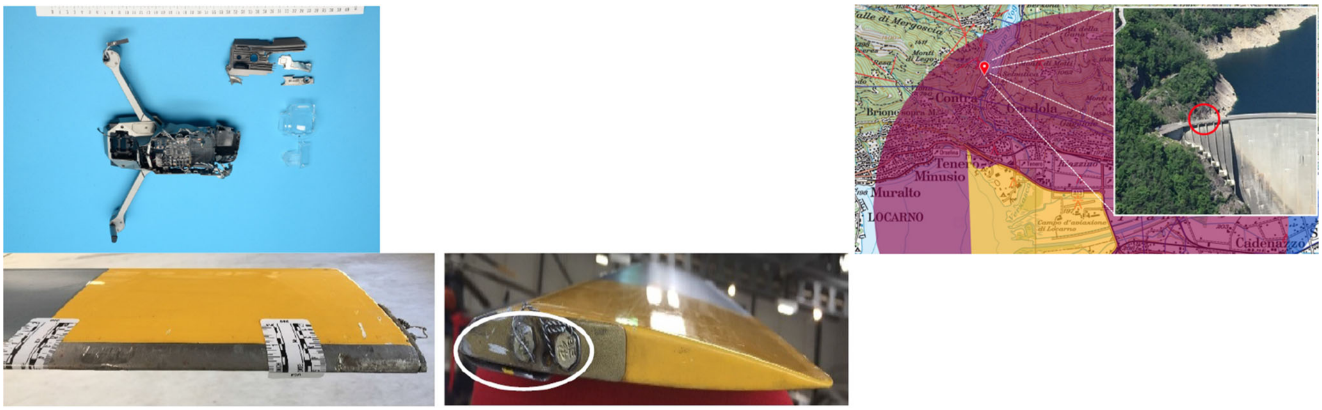

Fig. 15 Drone collides with GC2 helicopter

### 7 Legal Consequences of the Mid Air Collision

A collision with a manned aircraft may result in consequences for the UA operator. These may involve criminal liability for non-compliance with air traffic regulations and civil liability for damage to property or personal injury (death, bodily injury or health disorder).

There are no special international or European regulations on UAS regarding criminal liability. However, one can find criminal provisions in national law. For example, in Poland, such regulations exist in the country's Aviation Law and Criminal Code which could apply to a drone operator as well. A drone user can violate air traffic regulations. In such a case art. 211 and art. 212 of the country's Aviation Law would apply.

Article 211 provides for several offenses for performing flights with aircraft, contrary to the provisions of the Act, and among others: anyone who operates a flight using an aircraft inconsistent with the required airworthiness or inconsistent with the limitations specified in the airworthiness certificate, anyone who performs a flight against the obligations regarding the conditions of use of the aircraft in the Republic of Poland or anyone who, despite the ban on the emission of a laser beam or light from other sources in the airspace areas, emits or causes the laser beam or light from other sources to be emitted in the direction of the aircraft in a way that may cause glare and consequently create a safety hazard to the aircraft or the health of the crew and passengers on board, is subject to a fine, restriction of liberty or imprisonment for up to one year. The same punishment is imposed on whoever, not fulfilling his duty, allows such acts to be committed. [7] As an example of infringement of this Article is when a person with VLOS flight license performs a BVLOS flight or when a person is flying a drone with outdated aero-medical examinations, a person is operating a drone under the influence of alcohol or drugs, a person is doing acrobatic with drone over residential or a cluster of people etc.

Article 212 provides for several offenses for violation of the provisions of the Act in the field of air traffic and among others: anyone who, when performing a flight with an aircraft violates air traffic regulations in force in the area in which the flight takes place or crosses the state border without the required permit or in violation of the permit conditions or anyone who, contrary to the provisions of the Act, uses signs and signals in traffic that are unrelated to this movement or in a way that could be misleading to air traffic service units or crews of aircraft, is punishable by imprisonment of up to 5 years. The same punishment is imposed on whoever, not fulfilling his duty, allows such acts to be committed. If the perpetrator acts unintentionally, he / she is subject to a fine, restriction of liberty or imprisonment for up to one year. [7] As an example of infringement of this Article is when a person is flying a drone in the CTR zone (for accuracy at a distance of eg. 2 km from the airport), who did not inform the relevant services about the intention to perform the operation (ASM 1 and TWR) and did not obtain proper flight conditions approval. Another example is when a person is using the drone to smuggle goods across the state border or a person is using a drone in the Restricted Area above National Park without the consent of its authorities.

Pursuant to art. 173 of the Criminal Code anyone who causes a disaster on land or water or to air traffic, and thereby endangers the life or health of many people, or property to a significant degree is liable to imprisonment for between one and 10 years. If the offender acts unintentionally he or she is liable to imprisonment for between three months and five years. If this act results in the death of a human being or in grievous bodily harm to many people, the offender is liable to imprisonment for between two and 12 years. If this act results in the death of a human being or in grievous bodily harm to many people, the offender is liable to imprisonment for between six months and eight years. According to art. 174 anyone who causes an immediate danger of a disaster on land or water or to air traffic is liable to imprisonment for between six months and eight years. If the offender acts unintentionally, he or she is liable to imprisonment for up to three years.

The issue of civil liability for damage caused by the use of unmanned aircraft can be considered from many aspects. In the case of passenger transport, we should analyze the provisions on air carrier (or UA operator) liability for damage, including death or bodily injury. Then, there is also the issue of air carrier (or UA operator) liability for damage caused to transported goods (cargo). And finally, unmanned aircraft operations may also result in damage affecting people and property on the ground (both bodily injury, death and damage to property).

An air accident involving a drone may cause damage to persons and property directly involved in a flight or in transport (passengers, luggage, goods, mail, etc.), as well as damage to third parties (persons and property) in the event of a collision with another aircraft or a collision on the ground. These are two independent regimes of liability. In the first case, if the parties are bound by a contract of carriage or another type of contract, it is assumed that liability arises from the lack of or undue performance of contractual obligations (contractual liability). However, in the second case, the parties are not bound by any contract and therefore a different liability arises – one that is the result of a violation of civil law (tort liability). [26–28].

There are no special international or European regulations for drones regarding civil liability either. Therefore the general rules of the national law on civil liability applies. In most of the countries for damages caused to third parties the general rules of liability of an operator of manned aircraft applies. For example in Poland Aviation Law Act of 2002 states in the Article 206 that liability for damage caused by the movement of aircraft is subject to civil law for liability for damage caused by the use of mechanical means of communication raised by the forces of nature. Thus, this Article refers to the Civil Code, and particularly to Articles 435 and 436. Article 435 (1) C.C. establishes the rule of strict liability for injuries caused by enterprises or establishments which are set in motion by natural forces like steam, gas, electricity, fuel. The enumeration is not exclusive and atomic energy should be included. The same liability is imposed by Article 435 (2) C.C. on establishments manufacturing or using high explosives, for instance, mines. The person who is operating the enterprise on his own account is liable on proof of injury caused by accident unless he/ she proves that the damage was the result of force majeure or incurred through the exclusive fault of the person injured or of a third party. Article 436 refers to traffic accidents caused by motor-vehicles. According to Article the liability depends on possession of the vehicle and not on ownership. The result is that in the case of theft, the owner of a car ceases to be liable. There are two important exceptions to this rule. Principles of liability based on fault are applicable when persons are transported gratuitously and in the case of a collision (see Art. 436 (2) C.C.). A gratuitous guest must prove the fault of the possessor (or his servant). In the case of a collision general principles decide how far the fault of each driver contributed to the damage. [29]

The liability of an operator is therefore strict and he cannot escape of it unless he proves that the damage was the result of a force majeure or incurred through the exclusive fault of the person injured or of a third party. [30]

Therefore, it is important for a drone operator to purchase insurance (third party liability insurance). The analysis of regulations concerning insurance of operations involving the use of unmanned aerial vehicles shows a variety of solutions in different countries. Uniform regulations exist only at an EU level, but only for unmanned aerial vehicles with a take-off weight above 20 kg Therefore, there is an obligation to purchase insurance, but only for aircraft over 500 kg MTOW (at least 750,000 SDR). Drones below this mass are not included in the regulation. Additionally, there may be a problem in identifying the operator responsible for causing an accident. [1]

Pursuant to the ordinance of the Polish Minister of Transport, owners of certain drone models are subject to the obligation to purchase civil liability insurance. It applies to drones weighing from 5 to 20 kg. Their operators must purchase a policy with an insurance sum of 3000 SDRs, or approximately EUR 16,000. If the weight of the drone does not exceed 5 kg and is only used for sports or recreational purposes, then operators are not obliged to purchase civil liability insurance. Taking into account the damage that can be caused by a drone and the costs associated with compensation, even the amount required by the obligatory third party liability insurance seems very low.

### 8 Conclusions

Numerous reports indicate that drones will gradually replace "traditional" (manned) aviation in such fields as aerial photography, search and rescue operations, transmission network monitoring or precise aerial application activity. [31] The costs of using unmanned systems are incomparable to those incurred through manned aviation. Various services can be provided both remotely, i.e. with an operator (pilot) remotely controlling a UA, or autonomously in a pre-programmed manner without an operator's (pilot's) intervention or with their role simply limited to system supervision. [32]

Unmanned aerial vehicles facilitate many areas of socio-economic life. Their use may contribute to saving people's lives (e.g. the use of drones in emergency medical services). Available literature classifies drone usage in terms of functionality and industry. Functional use includes: monitoring, control, protection, data collection, image and audio recording, radio signal propagation, data transfer propagation, cargo shipment. Industry use constitutes: transport and logistics (including intralogistics), construction, agriculture, search and rescue, meteorology, geodesy, hydrography and cartography. [1, 33]

A growing number of drone operations have resulted in a higher number of drone incidents and accidents – and these aren't just about close encounters between manned and unmanned aircraft. These also include incidents that result in damage or destruction of UA, i.e. air accidents exclusively involving UA. Meanwhile, the principles that guide air accident investigations developed over the last few decades remain virtually unchanged. They are a manifestation of what may be called a reactive approach to aviation safety.

The analysis of the regulations on reporting UAS-related incidents as well as statistics on reporting said events has led to the following conclusions.

First of all, current regulations on reporting UAS-related events (in the EU it is the EU Regulation No. 376/2014), focus on the perspective of manned aviation. As a result, the majority of reported events involve situations where a drone or a drone-like object was spotted by the crew of a manned aircraft. This raises the practical problem of objectively identifying the operators of said UAS. In some cases, there is also the issue of confirming whether the flying object in question was actually a UAS (a false-positive) [14].

Nonetheless, it is essential to report such events since they provide the basis for identifying this major threat of using UAS, i.e. the violation of controlled airspace. The ultimate goals are to prevent collisions between UAS and manned aircraft.

Based on the available data, scientific or institutional analyses are carried out, and the results of these can be used in developing new regulations to ensure a required level of safety

– in the case of UAS – for third-parties on the ground as well as people on board the aircraft.

It can, therefore, be reasonably concluded that reporting and analyzing UAS-related incidents allows for the assessment and development of strategies for integrating manned and unmanned aviation. Moreover, it plays a role in creating regulations for UAS, which enables the further development of UAS operations with the aim of ensuring the safety of third-parties as well as manned aviation. Controlling many of the risks associated with UAS operations is best accomplished as a part of regulatory process that allows them in the first place [12]. When those regulation are in place, reporting and – in some cases – analyzing incidents is essential to verify the effectiveness of safety nets in place.

There is, therefore, a solid reason for reporting and analyzing UAS-related events other than accidents – giving regulators access to objective data on air safety. There are, however, some main challenges related to reporting and analyzing UAS-related events.

Firstly, the lack of uniform standards regarding the drone-related occurrences, including which types of data should be included in the reports. It was already identified that regardless of whether the systems are mandatory or voluntary, a key issue is the lack of consistency within those databases. [34] Therefore, the harmonization of data reporting is a critical step, even within the existing systems. [20]

Secondly, no standards for analyzing drone-related occurrences have been adopted. There are regulations for specific UAS accidents and these have already been mentioned at the beginning of the article. These types of events, however, are few and far between. When it comes to occurrences other than accidents or incidents, it seems reasonable to establish at least some basic and unified rules for reporting, classification, and in some cases, analysis. Root cause analyses of recurring problems, such as losing control with a specific type of drone can be a good example. Corrective measures taken early enough may prevent a drone accident caused by a drone that lost control once again. [20]

Thirdly, at EU level, no specific safety data related to UAS incidents has been published. We can find some safety data only in Annual Safety Reviews (ASR) published by EASA for 2016 and 2017. However, it was only once (in ASR for 2017), when EASA indicated some priority key risk areas with regard to the drone operations. It was based on statistics provided:

– Aircraft upset caused by loss of control;
– Risk of collision in the air;
– Risk of collision with an obstacle on the ground.

Because of the unknown reasons, the data has not been published in the following EASA Aviation Safety Review editions (2018, 2019). Lack of commonly available data and analyses about drone-related incidents seems odd. Especially, if we take into account how many drone-related legislative processes has been launched by EASA in the last three years, including adoption of delegated acts published by European Commission in July 2019 [19], followed by the extension of the EU competencies in 2018 [18, 20].

Finally, the large number of drone-related incidents have been reported under applicable EU occurrence reporting legislation. However, under the existing reporting schemes, only the licensed professionals operating manned aircraft are obliged to report safety occurrences. Therefore, the development of the reporting schemes dedicated to UAS operators or pilots seems to be necessary. [20]

The issue of applying principles of reporting and investigating air accidents or serious incidents to collisions involving both manned aircraft and UA or solely UA also requires further analysis. The available reports clearly indicate different approaches to qualifying such events. [20]

It should also be highlighted that drones require refinement, especially in technological terms. It is reasonable to take action aimed at raising awareness amongst UAS users of the need to report incidents, as well as engaging UAS users in the investigative process which follows such occurrences.

Finally, one should remember of the legal consequences that stem from non-compliance with operational regulations, including criminal liability and the obligation to compensate a victim for damages caused by a drone operator. Third party liability insurance is not only an added security for UA operators, but also a guarantee of compensation for injured parties.

**Acknowledgements** Research financed by the National Science Centre, Poland. Project No 2017/27 /B/HS5/0008 "Unmanned Aircraft. A New Era in Aviation Law".

**Authors Contributions** Piotr Kasprzyk – Sections 1,2,3,4,5,7.
Anna Konert – sections 1,2,5,6,7.

**Funding** Article is the effect of a research grant financed by the National Science Centre, Poland. Project No 2017/27 /B/HS5/0008 "Unmanned Aircraft. A New Era in Aviation Law".

### Declarations

**Ethical Approval** 'Not applicable'.

**Consent to Participate** 'Not applicable'.

**Consent to Publish** 'Not applicable'.

**Competing Interests** 'Not applicable'.

**Open Access** This article is licensed under a Creative Commons Attribution 4.0 International License, which permits use, sharing, adaptation, distribution and reproduction in any medium or format, as long as you give appropriate credit to the original author(s) and the source, provide a link to the Creative Commons licence, and indicate if changes were made. The images or other third party material in this article are included in the article's Creative Commons licence, unless indicated otherwise in a credit line to the material. If material is not included in the article's Creative Commons licence and your intended use is not permitted by statutory regulation or exceeds the permitted use, you will need to obtain permission directly from the copyright holder. To view a copy of this licence, visit http://creativecommons.org/licenses/by/4.0/.

## Data Availability

All data are available on the websites of EASA, EUROCONTROL or ECCAIRS, including EVAIR Bulletin No 20, EASA ASR of 2017, EASA Drone Collision TF Report (2016), ECAIRS data published in PL CAA ASR for 2017, EASA ASR for 2016.
